# Advances in hepatic stem/progenitor cell biology

**DOI:** 10.17179/excli2014-576

**Published:** 2015-01-06

**Authors:** Stefaan Verhulst, Jan Best, Leo A. van Grunsven, Laurent Dollé

**Affiliations:** 1 Liver Cell Biology Laboratory, Vrije Universiteit Brussel (VUB), Brussels, Belgium

## Abstract

The liver is famous for its strong regenerative capacity, employing different modes of regeneration according to type and extent of injury. Mature liver cells are able to proliferate in order to replace the damaged tissue allowing the recovery of the parenchymal function. In more severe scenarios hepatocytes are believed to arise also from a facultative liver progenitor cell compartment. In human, severe acute liver failure and liver cirrhosis are also both important clinical targets in which regeneration is impaired, where the role of this stem cell compartment seems more convincing. In animal models, the current state of ambiguity regarding the identity and role of liver progenitor cells in liver physiology dampens the enthusiasm for the potential use of these cells in regenerative medicine. The aim of this review is to give the basics of liver progenitor cell biology and discuss recent results vis-à-vis their identity and contribution to liver regeneration.

## The Basics of Hepatic Stem/Progenitor Cell Biology

**Figure 1:**
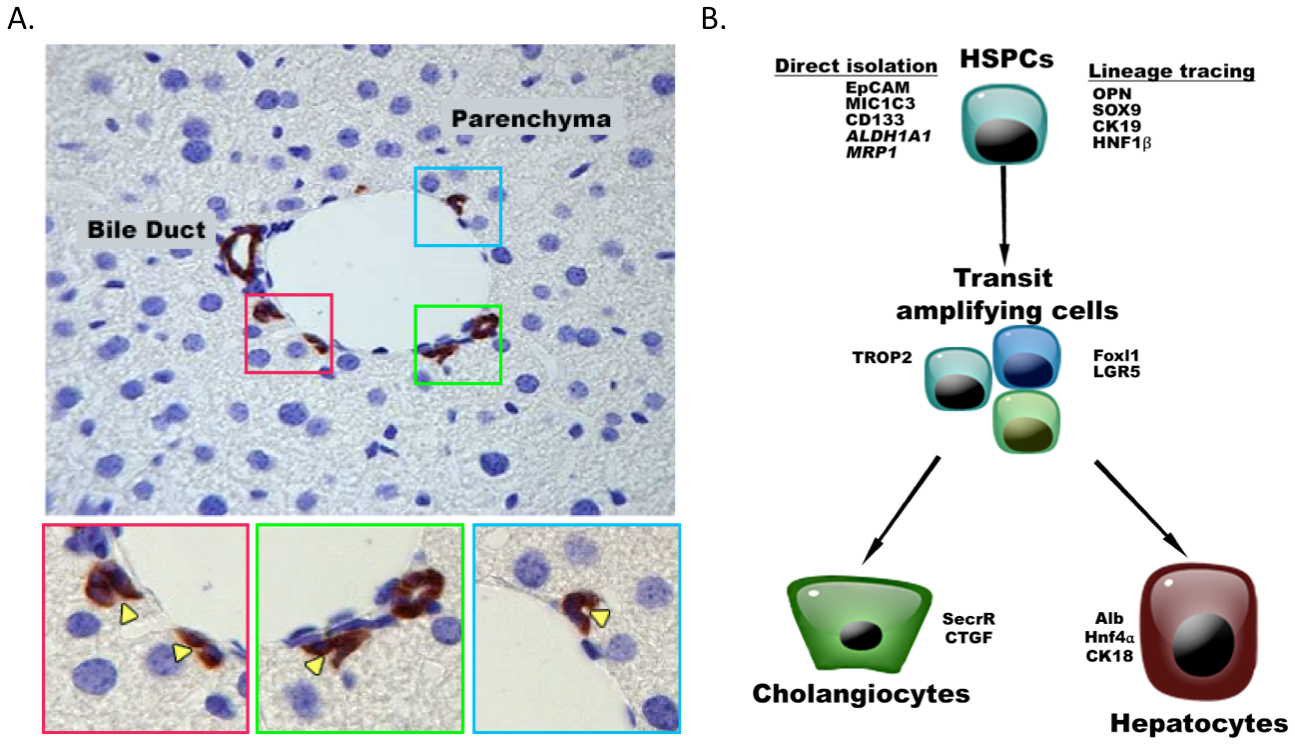
The niche of HSPCs and their expression markers (A) HSPCs are located in the smallest and most peripheral branches of the biliary tree, the ductules and canals of Hering. In healthy murine livers, canals of Hering are present in the portal area and consist of one hepatocyte and several cholangiocytes/HSPCs. By immunohistochemistry, cholangiocytes/HSPCs are identified by their Krt19-positivity (brown). Corresponding channels and their lumens are magnified in the lower panels (colored squares, yellow arrowheads indicate the lumen). (B) HSPCs can be isolated directly using FACS or MACS with antibodies directed to EpCAM, MIC1C3, CD133 or using a functional assay (ALDH1A1 (aldehyde activity), MRP1 (Side Population). When studying their functionality, lineage tracing of quiescent HSPCs is often used based on markers such as OPN, SOX9, CK19, HNF1β. Once activated, HSPCs become transit amplifying cells expressing the surface marker (TROP2) as well as Foxl1 and LGR5 which are also used for lineage tracing.

The liver has the amazing potential to regenerate when mild liver damage occurs. During this process, remnant resting hepatocytes will re-enter the cell cycle and efficiently replenish the liver through proliferation. A good example of the capacity of adult hepatocytes and bile epithelial cells to proliferate is seen during recovery from partial hepatectomy in rats and mice, when two-third of the liver is removed (Fausto et al., 2012[22]; Russo et al., 2011[63]). More importantly, this capacity is underlined by the ability to perform living-donor liver transplantation, where each half is capable of re-growing to support different individuals. During persistent and severe liver damage, hepatocytes no longer have the capacity to proliferate whereas hepatic stem/progenitor cells (HSPCs) are induced to expand, also known as a ductular reaction, oval cell reaction or oval cell hyperplasia. HSPCs exist in the smallest and most peripheral branches of the biliary tree, the ductules and canals of Hering (Kuwahara et al., 2008[43]; Theise et al., 1999[86]) (Figure 1A(Fig. 1)). Their niche is composed mainly of hepatic stellate cells, endothelial cells, Kupffer cells and a specific network of extracellular matrix (ECM) that retains all molecules secreted by the niche cells (Lorenzini et al., 2010[44]; Russo et al., 2011[63]; Van Hul et al., 2009[90], 2011[89]). Because of their self-renewal capacity, high proliferative ability and differentiation potential toward hepatocytes and cholangiocytes, HSPCs are considered as an attractive alternative source for liver cell therapy (Cantz et al., 2008[10]; Dan and Yeoh, 2008[15]).

Browsing of the literature describing the origin, fate and potential of HSPCs shows that investigators use different terms and abbreviations to describe the phenomenon of this liver cell type that has the above mentioned characteristics. Due to abbreviations and names such as oval cells, liver progenitor cells (LPCs), liver stem cells (LSCs), atypical ductal cells (ADCs), or intermediate hepatobiliary cells, it is sometimes difficult to deducewhether researchers are studying the same cell. While it is desirable to come to a nomenclature and classification of these -maybe different- cells, in this review we will use the term HSPCs to encompass the various liver stem/progenitor cell populations irrespective of species or injury model.

## Models to Study HSPCs

Liver regeneration can be broadly characterized into hepatocellular or biliary regeneration, which is dependent on the type of injury. Adaptive, but flexible crosstalk between the microenvironment (i.e. extracellular matrix (ECM) and neighboring cells, like Kupffer cells, myofibroblasts and hepatic stellate cells) and the stem-cells themselves are required to allow the activation of HSPCs (Boulter et al., 2013[7]). Different liver injury mouse models have been used to study this HSPC activation. Two commonly used diets, DDC (3,5-diethoxycarbonyl-1,4-dihydrocollidine) and CDE (choline-deficient, ethionine-supplemented), are employed to activate HSPC expansion and differentiation to hepatocytes or cholangiocytes. Indeed, the DDC diet results in an accumulation of protoporphyrin in hepatocytes leading to cholangitis (Fickert et al., 2007[24]; Preisegger et al., 1999[57]) whereas CDE resolves in hepatic damage with HSPC expansion (Akhurst et al., 2001[2]). Additional possibilities, like the use of a Methionine-choline-deficient (MCD) diet (Rinella et al., 2008[60]), intoxication by N-acetyl-p-aminophenol (APAP) (Kofman et al., 2005[41]) or the N-2-acetylaminofluorene (2-AAF) treatment in combination with 70% hepatectomy (mainly in rats) (Santoni-Rugiu et al., 2005[66]) are also frequently used to study HSPCs. In addition to the different methods used to stimulate HSPC proliferation/differentiation, researchers use different isolation procedures or transgenic lineage tracing markers making it virtually impossible to compare all the different experimental setups. The importance of careful extrapolation between individual species and the nature of the toxin-induced liver damage treatment was already highlighted in 2007 by a study of Jelnes and collaborators[36], showing remarkable phenotypic discrepancies exhibited by the progenies of the HSPCs in stem cell-mediated liver regeneration models between rats and mice. They used CK19-positivity (and additional markers, like AFP, MPK, and ABCG2) to evaluate the HSPC activation in different injury models and concluded that the CDE model is the most appropriate model to study this phenomenon and that C57Bl6 mice respond better than Fisher 344 rats (Jelnes et al., 2007[36]). Whether the same HSPC is activated differently due to the different nature of the injury or whether from the beginning different HSPC niches exist, which can be independently activated depending on the nature of the injury, are questions that largely remain unanswered.

## Characterization of HSPCs

**Figure 2:**
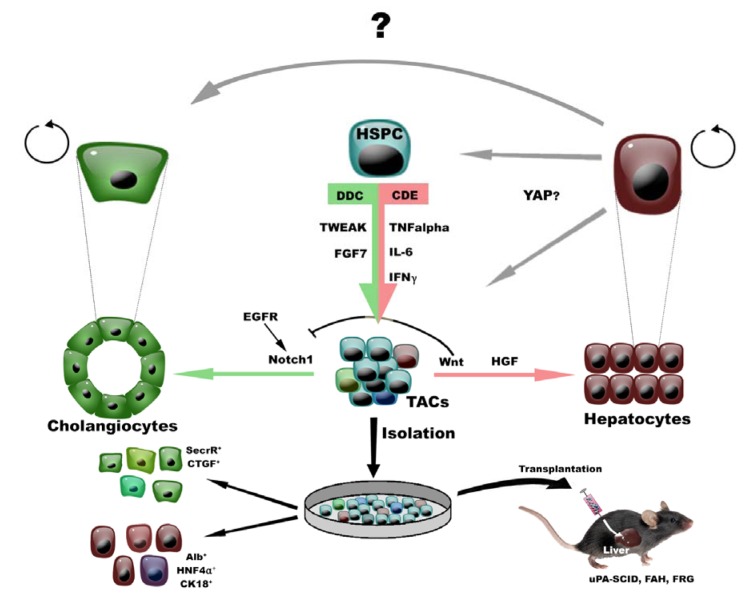
Regulation of HSPCs *in vivo* and *in vitro* In healthy livers, mature cells can autonomously replenish their hepatic compartment without intervention of HSPCs. Upon injury in rodent, artificially induced by specific diets (like DDC or CDE), HSPCs get activated resulting in their expansion, propagation in the parenchyma and potentially to liver repair. Many known cytokines and growth factors (like TWEAK, FGF7, TNFα, IL-6 or IFNγ) are partially responsible for their activation. Depending on the injury (cholangiocytic vs hepatocytic), transient amplifying cells (TACs) differentiate toward hepatocytes during DDC diet with the help of Wnt and HGF signaling. When CDE injury occurs, TACs preferentially differentiate into cholangiocytes using Notch1 signalization. Isolated HSPCs/TACs can be placed in culture dishes for in vitro expansion or differentiation toward cholangiocyte-/hepatocyte-like cells (bipotentiality) or can be transplanted to study their function in vivo in uPA-SCID (urokinase-type plasminogen activator-Severe combined immunodeficiency), FAH (Fumarylacetoacetase hydrolase) and FRG (FAH/Rag2/II2rg). The potential lineage conversion of hepatocytes to cholangiocytes and YAP-dependent transdifferentiation of hepatocytes to HSPC-like cells is indicated by grey arrows.

While the notion of HSPCs is widely accepted, many questions remain regarding their characteristics. This is partly due to the fact that specific markers for quiescent HSPCs still need to be discovered. On the other hand, several proteins or activities that discriminate the HSPCs from their surrounding cells exist; EpCAM- (Epithelial cell adhesion molecule, aka TROP1/ TACSTD1 (Yovchev et al., 2008[98])), Prom1- (Prominin1, aka CD133 (Rountree et al., 2007[62])) and MIC1-1C3- (macrophage inhibitory cytokine-1-1C3 (Dorrell et al., 2011[19])) antibodies or a combination of them, have been used to enrich these cells, while activities that are enhanced in HSPCs like efflux transporter activity (e. g. Side population technique (Govaere et al., 2014[29]) or aldehyde dehydrogenase activity (Dollé et al., 2012[18])) are more intricate but can also be used to isolate HSPCs (Figure 1B(Fig. 1)). Typically cells isolated by means of one of the above procedures are further characterized *in vitro* for their cell renewal and differentiation capacity or *in vivo* for their ability to repopulate an injured liver with HSPC-derived hepatocytes and cholangiocytes (Figure 2(Fig. 2)).

The problem with the aforementioned markers is that they are also expressed on regular biliary epithelial cells. The current view is that, once awakened by signals from the surrounding liver tissue, HSPCs become transit amplifying cells expressing some proteins not expressed by regular biliary epithelial cells such as Foxl1 (Forkhead Box l1), TACSTD2/Trop2 (Tumor-associated calcium signal transducer 2) or LGR5 ((leucine-rich-repeat-containing G protein-coupled receptor 5) (Huch et al., 2013[33]; Okabe et al., 2009[54]; Sackett et al., 2009[64]). Foxl1 and LGR5 transgenic mice have been successfully used to isolate these transit-amplifying cells from injured livers for further expansion and characterization, but have not been compared with each other yet. We are not aware of TACSTD2 transgenic mice, which would allow the comparison between Foxl1, LGR5 and TACSTD2 expressing cells from mice exposed to similar injuries with respect to their activation-, differentiation- and proliferation potential. To carry out such a study in one lab would require quite some resources, but could reveal whether we can actually compare results obtained using different HSPC lineage tracing markers.

## HSPCs Signaling Pathways

Understanding the signaling pathways that are involved in HSPC maintenance, activation and differentiation are of great interest to those working in the field of liver regeneration. Not only can these pathways potentially steer the *in vitro* culture/expansion of HSPCs but it could also give clues on how to direct these cells in injured livers to perhaps contribute to liver regeneration. Inflammatory cytokines play an important role in HSPC response. The first cytokine described that has an explicit impact on HSPCs is tumor necrosis factor (TNF)-like weak inducer of apoptosis (TWEAK), which is produced by monocytes, T lymphocytes and macrophages whose expression increases in contexts of acute injury, inflammatory disease and cancer. The expression of its receptor Fn14, normally expressed by epithelial and mesenchymal cells, is also relatively low in healthy tissue but is dramatically induced in injured and diseased tissue, such as in livers following a DDC treatment. HSPC activation by DDC was significantly reduced in Fn14-null mice and by the use of an anti-TWEAK antibody while overexpression of TWEAK in hepatocytes (Jakubowski et al., 2005[35]) or exogenous TWEAK administration leads to periportal oval cell hyperplasia (Bird et al., 2013[5]). Recently, Miyajima's group found evidence that FGF7-dependent HSPC activation effectively contributes to progenitor-dependent liver regeneration and survival in severe liver injury (Takase et al., 2013[81]). Indeed, FGF7 expression was induced concomitantly with a HSPC response in the liver of mouse models (such DDC, CBDL), as well as in the serum of patients with acute liver failure corroborating earlier data (Dezso et al., 2007[17]; Murakami et al., 2011[52]; Steiling et al., 2004[78]). FGF7-deficient mice exhibited markedly repressed HSPC expansion and higher mortality upon toxin-induced hepatic injury. Furthermore, transgenic expression of FGF7 *in vivo* led to the induction of cells with features of HSPCs and improved hepatic dysfunction (Takase et al., 2013[81]). Obviously it cannot be excluded that FGF7 may have a protective effect on damaged mature cells (i.e. hepatocytes and cholangiocytes) or even initiates transdifferentiation of damaged -or surrounding- hepatocytes (see below). Although the HSPC response in Fn14-deficient mice was attenuated after CDE treatment, it was restored later on and ultimately resulted in a level similar to that in WT mice (Tirnitz-Parker et al., 2010[87]). Contrarily, in FGF7-deficient mice, HSPC activation was never induced even after long-term liver injury (Takase et al., 2013[81]). This suggests a more direct role of FGF-7 in HSPC induction, while that of TWEAK maybe rather enhancing and not necessarily indispensable. 

Many additional factors have been reported to be involved in HSPC activation and probably also steer their differentiations. Hepatic growth factor (HGF)/c-MET and Epidermal growth factor (EGF)/EGF receptor (EGFR) are key regulatory elements to determine HSPC activation and differentiation. In c-MET knockout mice, HSPC response was significantly less upon DDC injury *in vivo *(Ishikawa et al., 2012[34]) suggesting that HGF is important for HSPC activation. On the other hand EGFR seems to be necessary for differentiation of HSPCs, since stimulation of the EGFR triggers the Notch1 pathway resulting in cholangiocyte differentiation and concomitant inhibition to the hepatic lineage (Kitade et al., 2013[39]). Wnt3a is expressed by macrophages triggered by hepatocyte damage (CDE diet) and subsequent debris engulfment, leading to the inhibition of Notch in HSPCs and hepatocyte regeneration, while biliary damage activates Notch in HSPCs resulting in biliary specification. Interestingly, the depletion of macrophages during hepatocellular damage results in Notch activation, instead of Wnt signaling, thereby favoring biliary specification (Boulter et al., 2012[6], 2013[7]). Other essential cytokines, like TNFα, interferon gamma (IFNγ), interleukin 6 (IL-6) and oncostatin (OSM), can also stimulate HSPC activation and were already described in earlier studies in which researchers were comparing partial hepatectomy versus stem cell-mediated liver regeneration (Knight et al., 2005[40]; Shiojiri 1997[75]; Yeoh et al., 2007[96]; Znoyko et al., 2005[101]) (reviewed in (Kang et al., 2012[38])).

## Origin of HSPCs

The origin of HSPCs is still under debate. HSPCs and cholangiocytes share similar molecular markers suggesting that HSPCs originate from the biliary tree. It is still being disputed whether all cholangiocytes or merely a subpopulation are precursors of HSPCs or whether a specialized cell type is the origin of HSPCs. Although places like intralobular bile ducts and the parenchymal-stromal interface have been described as HSPCs niches (Kuwahara et al., 2008[43]), the canal of Hering is the most accepted HSPC niche (Paku et al., 2001[55]; Theise et al., 1999[86]) (Figure 1A(Fig. 1)); anatomically it is the most logical area because cholangiocytes and hepatocytes meet each other in this region. During early liver development (E9-11), Dlk1^+^/EpCAM^+^ cells are defined as hepatoblasts expressing albumin and they are capable of differentiating into hepatocytes (EpCAM^-^/Dlk1^-^/Alb^+^/CK19^-^) and cholangiocytes (EpCAM^+^/Dlk1^-^/Alb^-^/CK19^+^) (Fausto et al., 2012[22]; Tanaka et al., 2009[82]). Only one study showed that HSPCs emerge from the progeny of the ductal plate cells. Carpentier and colleagues elegantly showed that when SOX9-Cre ER^T2 ^;ROSA26R^YFP^ offspring were exposed to DDC- or CDE diets at 4 weeks after birth, YFP positive cells expanded which were derived from Sox9 positive ductal plate cells at day E15,5 (time of tamoxifen injection). These ductal plate derived YFP positive cells co-expressed SOX9, OPN and other oval cell markers (Carpentier et al., 2011[11]).

## Participation of Endogenous HSPCs in Liver Repair

Many studies have shown the potential of stem cells or isolated HSPCs to rescue a damaged liver when HSPCs have an advantage over damaged hepatocytes in mouse models such as the uPA/SCID and FAH or FRG mouse models (Brezillon et al., 2008[8]; Grompe et al., 2013[30]) (Figure 2(Fig. 2)). Until recently, the actual contribution of endogenous HSPCs to the recovering liver mass was not well documented. Two very elegant lineage tracing studies using inducible OPN-Cre and HNF1β-Cre transgenic mice to follow the fate of HSPCs gave some insight. They demonstrated that during or after liver damage, and only in the CDE diet, merely a small percentage of hepatocytes is derived from HSPCs (between 0.8-2.5 %) (Espanol-Suner et al., 2012[21]; Rodrigo-Torres et al., 2014[61]). In these reports, HSPCs did not give rise to hepatocytes during homeostasis or liver injury caused by BDL, DDC, PH and CCl_4_. This was in contrast to a later study that showed that lineage tracing of LGR5-CRE positive cells yielded hepatocyte-like cells after one single injection of CCl_4_ or the DDC and MCDE diets (Huch et al., 2013[33]). None of the studies found a contribution of HSPCs to hepatocyte turnover in healthy animals. This compelling data has been undermined by several studies showing that there is no contribution of HSPCs in any of the situations described above. Instead of following the fate of HSPCs, the fate of hepatocytes in these studies is traced using either infection by AAV-Ttr-Cre viruses (Malato et al., 2011[45]; Schaub et al., 2014[67]) or regular Mx1-Cre mice (Tanimizu et al., 2014[83]) and applying different liver injuries. The use of Adeno Associated Viruses (AAV) expressing Cre recombinase in a cell type-specific manner eliminates the use of Tamoxifen administration and guarantees high recombination efficiency. Using this approach they show that there is probably no contribution of HSPCs but that rather the hepatocytes were the source of the repopulating hepatocytes, again in all possible injury settings. One can think of several explanations when trying to elucidate the discrepancy between these studies. As mentioned before, the use of different strains and age of the mice, injury models and technical approaches to investigate the contribution of HSPCs to hepatocyte regeneration makes the comparison more difficult. More importantly, the majority of HSPC tracing studies use Tamoxifen-inducible Cre-lines to carry out lineage tracing (Metzger, 2001[47]; Feil et al., 2009[23]). Several papers have highlighted some drawbacks of the use of this system in liver or HSPC tracing studies. First of all, the half-life of Tamoxifen in mice is rather long (T_1/2_=5-7 days) (DeGregorio et al., 1989[16]) and traces of the active compound can be found up to 4 weeks in mice (Reinert et al., 2012[59]). If the liver injury is induced within this time frame one can expect that recombination in a small number of cells expressing the CRE can occur; these can be newly formed cells or cells previously not expressing the transgene of interest. Secondly, Tamoxifen itself can induce the expression of genes that are used to drive the CRE expression. Both shortcomings are exemplified when using Sox9-CreER^T2^ mice to trace biliary epithelial cells/HSPCs (Furuyama et al., 2011[27]). Carpentier et al. (2011[11]) showed that Tamoxifen injection in healthy mice induced ectopic Sox9 expression in hepatocytes already within 18 hours, while Yanger et al. (2013[95]) showed that DDC-induced liver injury induces the expression of Sox9 in hepatocytes. Hence, the labeling observed in hepatocytes using Sox9-CreER^ T2^ mice (Furuyama et al., 2011[27]) is likely not only due to differentiation of Sox9 positive biliary cells but rather demonstrates that hepatocytes (re-)express Sox9 when injured or treated with Tamoxifen. Thirdly, the recombination efficiency achieved by the AAV8-based methods is extremely high reaching an average of 99 % in all injury settings according to the latest reports (Malato et al., 2011[45]; Yanger et al., 2014[94]). The recombination efficiencies reached by the traditional transgenic lineage tracing is much lower varying from ~ 69.1 % in OPN- (Espanol-Suner et al., 2012[21]), ~ 28.7 % in Hnf1b- (Rodrigo-Torres et al., 2014[61]), ~ 10.5 in CK19- (Schaub et al., 2014[67]) to 7 % in Sox9-CreER mouse lines (Tarlow et al., 2014[84]); the first two reports show a contribution of HSPCs to hepatocyte repopulation whereas the last two reports failed to show this. Perhaps a certain efficiency is needed in order to demonstrate a contribution of such a rare cell population.

An alternate hypothesis that might at least partially explain the obtained results from these studies, is that the low hepatocyte repopulation from HSPCs is a matter of hepatobiliary linkage but not of massive hepatocyte production (Theise et al., 2013[85]). In this scenario, damage of peribiliary hepatocytes, which are linked to the adjacent very small cholangiocytes (putative stem cells) of the Hering channel, would trigger HSPC activation and differentiation towards such a peribiliary hepatocyte. These hepatocytes must have a specialized surface that allows them to link with these tiniest cholangiocytes with cell adhesion molecules that are distinct from those required for linking to other hepatocytes (Gouw et al., 2011[28]). By producing these adjacent hepatocytes, the HSPCs restore the link between hepatocyte canaliculi and the biliary tree, allowing for free bile flow. Hepatocyte repopulation is thus low only when compared with total hepatocytes mass. But if one considers the peribiliary, channel of Hering-associated hepatocytes as a specialized subcompartment, then the repopulation of that subcompartment may in fact be quite robust (Theise et al., 2013[85]).

Another hypothesis that has been tested in mouse models of liver injury is that lineage conversion leads to *de novo* formation of mature hepatobiliary cells (Figure 2(Fig. 2)). Lineage conversion or trans-differentiation is a process where one mature adult cell transforms into another mature adult cell (Michalopoulos, 2011[48]; Suzuki, 2013[79]). The hypothesis is based on observations from human pathology; biliary markers (Krt19, HNF1β, HNF3α, and HNF3β) were detected in human hepatocytes in several cholangiopathies (Shin et al., 2011[74]). In mice, several reports show that under certain conditions (more particularly in cholangiocytic damage settings), hepatocytes can adjust their cellular program to biliary epithelial cells (Michalopoulos et al., 2005[50]; Sekiya and Suzuki, 2014[72]; Tanimizu et al., 2014[83]; Yanger et al., 2013[95]). Two pathways implicated in this transition are Notch and Hippo signaling. Evidence for these pathways comes from overexpression studies using again AAV-Cre transduction leading to overexpression of either the N-terminal intracellular Notch domain (NICD) (Yanger et al., 2013[95]) or the Hippo effector YAP (Yimlamai et al., 2014[97]). Overexpression of NICD in hepatocytes by using AAV-TBG-Cre induced an “intermediate” phenotype characterized by coexpression of OPN, Sox9 and HNF4a (Yanger et al., 2013[95]). YAP expression is high in the bile duct and expressed at lower levels in hepatocytes under normal conditions. Overexpression of constitutively active YAP in hepatocytes using AAV-TBG-Cre leads to a morphological change of hepatocytes into small oval cells that express K19, SOX9 and MIC1C3 (Yimlamai et al., 2014[97]). Furthermore YAP needs Notch signaling since deletion of the RBP-J effector of Notch signaling abolishes this YAP-mediated transdifferentiation (Yimlamai et al., 2014[97]). With respect to efficient recovery of liver damage and survival, lineage conversion does have advantages; the existence of various cellular sources, potentially able to provide any cell-type, any time and in sufficient number, allows for quick recovery in situations that are critical for the survival of the organ (Michalopoulos 2014[49]; Tanimizu et al., 2014[83]). Besides their seemingly modest - or no - contribution to repopulate the liver, HSPCs must have a function in the recovering liver, such as perhaps giving instructive cues to surrounding niche cells or recovering/dedifferentiating hepatocytes. To this end, modulation of the HSPC compartment appears a more realistic option in the treatment of hepatic failure than cell transplantation, since the latter is anyway compromised due to the damaged / bad infrastructure of an injured liver that irrevocably affects the potential effect of the transplanted cells. One can imagine that tissue regeneration may occur either by engraftment and differentiation of the donor cells at the site of injury or by paracrine mechanisms that stimulate endogenous HSPC pools to contribute to initiation of tissue repair. This hypothesis has been confirmed for some types of stem cells (Fouraschen et al., 2012[25]; Parekkadan et al., 2007[56]; Woo et al., 2012[92]; Zagoura et al., 2012[99]). In favor of such theory, some reports point out the additive value of individual or combined trophic factors (TWEAK, SCF, GM-CSF) as direct modulators of liver repair in mice with acute hepatic injury (Meng et al., 2012[46]; Swenson et al., 2008[80]; Tirnitz-Parker et al., 2010[87]). Thus far, no regenerative or survival signals have been attributed to the secretome of HSPCs.

## Enteric Dysbiosis and HSPCs Activation?

Following the acquisition of multicellularity, organisms with increasing levels of specialized cells, tissues and organs emerged during evolution. To coordinate these specialized organs, long-distance interorgan communication networks appear to directly converse their states to one another. An illustration is the co-evolution of the gut microbiota with its host in a carefully balanced system in which each requires the other (Holmes et al., 2012[32]; Nicholson et al., 2012[53]). If, however this balance is perturbed (dysbiosis) this potentially predisposes the host to a number of diseases marked by an aberrant common immune responses including inflammatory bowel disease, asthma, obesity, liver triglyceride storage, insulin resistance, metabolic syndrome and cancers (Cani, 2014[9]; Zhao, 2013[100]). The past decade has witnessed an explosion in studies examining the relationship between the microbiota and human health through the existence of inter-organ communication networks, in which gut microflora-fat tissue (Munukka et al., 2014[51]) or gut microflora-liver (Fouts et al., 2012[26]; Quigley et al., 2013[58]; Yan et al., 2011[93]) axes are described. It is now accepted that gut microbiota contribute to the management of energy homeostasis, glucose metabolism and inflammation-mediated metabolic diseases (Cani, 2014[9]; Serino et al., 2014[73]). Such studies have yielded a general hypothesis whereby microbiota products activate the innate immune system to drive pro-inflammatory gene expression thus promoting chronic inflammatory disease of the liver (Chassaing et al., 2014[12]).

**Figure 3:**
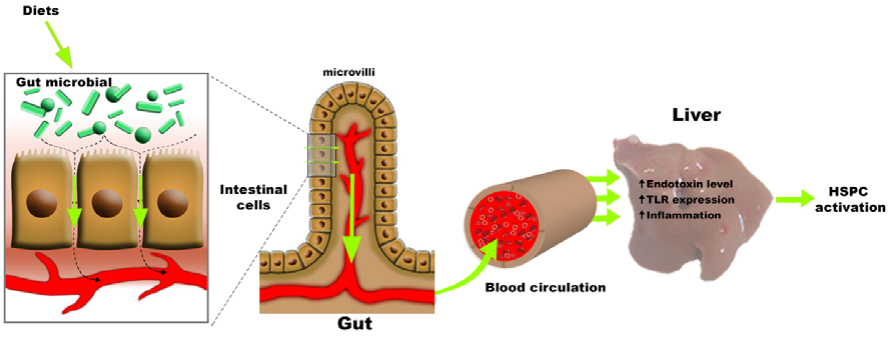
Enteric dysbiosis and HSPCs activation. Changes in the gut microbiota due to dietary input leads to alterations in the integrity of the intestinal barrier resulting in enhanced permeability and facilitates the translocation of bacteria or their products into the circulation. Green arrows illustrate how this dysregulation of the gut microbiota might influence HSPC activation.

Although we start to understand the process of liver regeneration, what initiates it is not entirely understood. Without any doubt, a common determinant is inflammation, which is characterized by the activation/recruitment of macrophages, concomitantly followed by myofibroblasts activation, along with the deposition of different kinds of extracellular matrices as supportive material. One can imagine that deregulation of the gut microbiota could contribute to the initiation or differential mode of regeneration, i.e. HSPCs driven or not (Figure 3(Fig. 3)). Many arguments emerging from the literature support a role for the gut microbiota in liver disease (for reviews see (Abu-Shanab and Quigley, 2010[1]; Chassaing et al., 2014[12]; Quigley et al., 2013[58]; Schnabl, 2013[69]; Sekirov et al., 2010[71]) and the special issue of Gastroenterology, 146 (6) 2014): 

(i) An overarching mechanism by which an aberrant microbiota negatively impacts health is by driving chronic inflammation (Abu-Shanab and Quigley, 2010[1]; Chassaing et al., 2014[12]; Quigley et al., 2013[58]; Schnabl, 2013[69]); 

(ii) Gut-derived endotoxins play a central role in the initiation of acute liver injury and progression to chronic liver disease (Chassaing et al., 2014[12]); 

(iii) An imbalanced intestinal homeostasis results in a breach of the gut barrier and subsequent microbial translocation (Fouts et al., 2012[26]); 

(iv) Selective intestinal decontamination with antibiotics is beneficial for patients and prevents experimental liver injury (Cirera et al., 2001[13]); 

(v) Mice with genetic deletions in the lipopolysaccharide (LPS) signaling pathway are resistant to experimental liver injury and fibrosis (Hartmann et al., 2012[31]; Seki et al., 2007[70]; Yan et al., 2011[93]); 

(vi) Transplantation of microbiota from diseased mice to germfree mice transfers some aspects of diseased phenotypes, indicating that altered microbiota plays a role in disease establishment and manifestation (Chassaing et al., 2014[12]; Sekirov et al., 2010[71]). 

Recently, Fouts and collaborators[26] have investigated dynamics of bacterial translocation and changes in the enteric microbiome in early stages of liver diseases (Fouts et al., 2012[26]). Cholestatic liver injury was induced by ligation of the common bile duct (CBDL) and toxic liver injury by injection of carbon tetrachloride in mice. Increased intestinal permeability and bacterial translocation occurred one day following liver injury in both disease models. The qualitative changes in the intestinal microbiome were investigated and the data revealed that minor changes were noticed following CBDL, while CCl_4_ administration resulted in a relative abundance of Firmicutes and Actinobacteria compare with oil-injected mice (Fouts et al., 2012[26]). Thus, the changes in enteric microbiome differ depending on the nature of the (induced) liver disease in mice. Since all diets inducing artificially the HSPC compartment (CDE, DDC, MCDE) have the obligatory passage through the gut in common, one can imagine that these dietary treatments have an impact on the gut microbiota, thereby also influencing the HSPC response (Figure 3(Fig. 3)).

## Improve and Fine-Tune the in vitro Strategies?

Many research efforts are focused on ways to expand hepatocytes or have perfect differentiation conditions of stem cells towards functional hepatocytes. Both are not current practice; hepatocytes in culture lose their functionality very quickly while stem cell differentiation towards hepatocytes often only reaches the hepatocyte-like cell level (Sancho-Bru et al., 2009[65]). Current methods for differentiation of HSPCs involves often mixtures of soluble growth factors and/or cytokines (HGF, EGF, FGF-1/2/4/7, SCF, TGF_α__,__β_) (Dollé et al., 2012[18]; Snykers et al., 2009[77]; Takase et al., 2013[81]) and many additives such as, dexamethasone, sodium butyrate, insulin, transferrin, nicotinamide and hydrocortisone (reviewed in Snykers et al., 2009[77]) (Figure 2(Fig. 2)). The expansion of HSPCs is not an easy task; cells tend to 'spontaneously' differentiate towards hepatic lineages, hampering efficient amplification while preserving the HSPC features. Early work by the lab of Lola Reid reported on efficient clonogenic expansion of human adult EpCAM^+^-HSPCs using a serum-free, defined medium (Kubota medium (Kubota and Reid, 2000[42])). The self-renewal capacity of HSPCs was indicated by phenotypic stability after expansion for up to 150 population doublings in this Kubota medium, with a doubling time of 36 h, allowing expansion of HSPCs for 6 months (Schmelzer et al., 2007[68]). Currently, the culture method that employs organoid structures embedded in Matrigel in the presence of R-Spondin seems to be the most efficient way of expanding LGR5^+^- or MIC1-1C3^+^/CD133^+^/CD26^-^HSPCs (Dorrell et al., 2014[20]; Huch et al., 2013[33]). These cultures maintain a stem cell phenotype and allow for differentiation towards hepatocytes and cholangiocytes (Huch et al., 2013[33]). The recent *in vivo* findings, highlighting the importance of Wnt, Notch, FGF7 and Tweak signaling, have thus far not been extrapolated to improve *in vitro* differentiation methods of HSPCs (or other stem cells) into hepatocytes. 

*In vivo*, HSPCs are not only influenced by growth factors and cytokines, changes in the extracellular matrix (ECM) seem to effect the proliferation and differentiation as well. The ECM is a complex structure made up of protein fibers that serve as a dynamic substrate that supports tissue repair and regeneration (Baptista et al., 2011[4]; Crapo et al., 2011[14]). Changes in Collagen (Espanol-Suner et al., 2012[21]; Kallis et al., 2011[37]; Van Hul et al., 2009[90]) and Laminin (Lorenzini et al., 2010[44]; Espanol-Suner et al., 2012[21]) deposition clearly weigh on the proliferation and differentiation capacity of activated HSPCs in rodents and humans. Mimicking these 3D changes in ECM in a dish has become possible by the development of decellularization techniques (Badylak et al., 2009[3]; Shupe et al., 2010[76]). Uygun et al. (2010[88]) successfully decellularized a recellularized liver matrix with primary rat hepatocytes, allowing culture for 48 hours. A parallel study using a similar approach reported on the efficient seeding and culture of human fetal liver cells and human umbilical vein endothelial cells for 7 days (Baptista et al., 2011[4]).

Similar decellularized matrixes also increased hepatocyte differentiation of human fetal HSPCs when used as a coating substrate for regular tissue culture plates (Wang et al., 2011[91]). Clearly, if these novel techniques evolve even further they might enable reproducible expansion and differentiation of HSPCs in the near future.

## Conclusion

In these last couple of years we have learned much about the pathways and conditions involved in HSPC activation thanks to sophisticated genetically modified mouse models. These same models are currently in conflict about the existence and function of HSPCs during liver injury and regeneration. Perhaps novel mouse liver injury models, more representative of human disease, need to be developed to fully unravel the existence, identity and function of HSPCs.

## Current Grant Funding

LD & LvG: Interuniversity Attraction Poles (IAP) - phase VII - contract P7/47 (Federal Science Policy -BELSPO) (10/2012-09/2017); SV: Flemish Government Agency for Innovation by Science and Technology, IWT/SB/121548 (2013-2016).

## Notes

Leo A. van Grunsven and Laurent Dollé contributed equally to this publication.
